# Swedish preschool teacher’s preferences and experiences of an applied learning intervention to support children with ESSENCE symptoms

**DOI:** 10.1186/s12991-026-00666-1

**Published:** 2026-05-17

**Authors:** B. M. Gustafsson, M. Sund Levander

**Affiliations:** 1 Department of Child and Adolescent Psychiatry, Department of Biomedical and Clinical Sciences, Linköping University, Linköping, Sweden; 2 Department of Psychiatry and Rehabilitation, Sweden and Child and Adolescent Psychiatry Clinic, Region Jönköping County, Jönköping, Sweden; 3 Department of Health, Medicine and Caring Sciences, Medical Faculty, Linköping University, Linköping, Sweden

**Keywords:** Applied learning, ESSENCE symptoms, Intervention, Interviews, Preschool teachers, Questionnaire

## Abstract

The purpose was to study preschool teachers’ preferences and experiences of an applied learning intervention to support children with ESSENCE symptoms. Preschool teachers (*n* = 164) filled out a questionnaire about their experiences, and 20 participated in individual interviews about preferences and experiences of the intervention. We used descriptive statistics for the questionnaire and inductive qualitative content analysis to analyze the interviews and free text answers in the questionnaire. The results showed that 74% experienced that they increased their understanding of the child’s difficulties, while 48% gained ideas that could be helpful in managing children. Specifically, the respondents emphasized the value of talking and reflecting together with guardians about a specific child. To share experiences from different contexts provided an understanding of the challenges the child, and the adults around them, faced in daily life. It also provided insight into the child’s opportunities, abilities and resources. The qualitative latent analyses revealed that the preschool teachers’ expectations corresponded well to their experiences after completing the training. The main categories *Collaboration between guardians and preschool teachers* and *Professional knowledge about children with special needs* describes their needs for supporting children with neurodevelopmental symptoms. Together they form the theme of a child-centered approach. In conclusion, the present findings add insights about collaborative learning, application of professional knowledge, and guardians and preschool teachers sharing experiences and reflecting together to support the child. In this way, the study contributes to the improvement and application of Child and Family Centered Care in practice.

## Background

Children with neurodevelopmental symptoms are at greater risk of mental illness later in life [28, 37, 38], and face an increased risk of impaired quality of life, failure at school, crime, and social exclusion [7, 20, 25, 47, 57, 58].The Swedish preschool is a context in which neurodevelopmental symptoms can be identified and good mental health promoted [2, 25]. As part of the ongoing project Mental Health, Learning, Development, and Collaboration for Young Children (PLUSS), this paper investigates Swedish preschool teachers’ preferences and experiences relating to an applied learning intervention to support children with neurodevelopmental symptoms.

Early Symptomatic Syndromes Eliciting Neurodevelopmental Clinical Examinations (ESSENCE) is an umbrella term for early-onset developmental neurological problems that largely overlap. ESSENCE highlights comorbidity and includes clinical symptoms displayed by preschool children in relation to general development, behaviour, attention, activity, social interaction, communication and language, motor coordination, and mood and/or sleep [25]. About 13% of Swedish boys and 7% of girls display identifiable ESSENCE symptoms affecting their functioning in everyday life ([25], [26]). Approximately 85% of Swedish children aged 1–5 and 95% of children aged 4–5, including children with.

Almost 87% of all children in Sweden aged one to five years old are enrolled in preschool. The majority of the preschools, 70% are run by non-profit municipal entities [53]. Swedish preschools are designed to create a safe environment where children are mentally stimulated and can improve their interactions with peers and teachers and general socialization outside the family unit. Compared to others, children attending preschool are privy to better health and life chances, as well as to social, emotional, and cognitive development, including intellectual abilities such as information processing, problem solving, language development, memory, and the ability to experience, regulate, and express emotions and explore their world [10, 11, 33, 49].

The preschools provide a unique context for supporting the development of children with social-communication challenges [49]. Preschool teachers meet both the children and the guardians daily. They see the child in everyday functions together with other children and have pedagogical knowledge about child development. The engagement and social interactions that take place in the Swedish preschool context are important factors in promoting development, learning, and mental health among young children [27, 40]. Hence, preschool is a context where neurodevelopmental symptoms can be identified early on [3], and where good mental health can be promoted ([2]; Gustafsson & Sund Levander, [31]). Professionals in Swedish preschools include preschool teachers with a university degree and other staff who have training equivalent to preschool teachers and/or experience of promoting children’s development and learning. Special educators have the responsibility of supporting the management of obstacles in children’s everyday life (Lpfö, [41]). Hereafter, all respondents in the present study are referred to as preschool teachers.

According to Swedish standard (Lpfö, [41]), preschool teachers are responsible for children’s learning and continuing development, but this refers to the preschool activities themselves and not the individual child. Preschool teachers should also aim to cooperate well with parents and offer developmental assessment meetings at least once a year. Preschool should be inclusive and inequalities in society compensated for through safe care and education. This means that every child should receive the support and care that they need from their preschool teacher. The preschool teacher’s care offers each child an emotional relationship, security, consolation, and protection. Early detection consists both of identifying children who need further support in addition to what is provided to all children, and of teachers reporting to external specialists when children in need are detected. Detection is primarily discussed as reporting children at risk to external experts. Children need to feel safe in order to be able to engage in both play and education. The idea of receiving a good standard of care needs to be incorporated into this. Since a revision of the curriculum in 2010, the preschool assignments of conveying knowledge and learning have become somewhat clearer (Lpfö 98, 2019). The Swedish preschool curriculum stresses the need to consider every child’s engagement when it comes to daily activities ([2]; Lpfö 98, 2019). Swedish preschools are somewhat unique in relation to other countries outside Northern Europe as far more time is spent in free play rather than instructed learning tasks, and children often spend a lot of time in free play outdoors [22, 43]. Higher levels of education among preschool teachers seem to have positive effects on the interactions between teachers and children as well as the children’s cognitive and social development [16, 19].

Current research has expanded our understanding of neurological developmental processes and the impact of parenting on children’s school readiness [8], For children with ESSENCE symptoms there are different interventions described intended to promote children’s social interaction and emotional and behavioural development [18, 51]. Justicia-Arráez et al., [36], reported positive effects of preschool teachers promoting social-emotional learning in preschools on emotional and communication skills, prosocial behaviors, problem-solving, and social interaction. Yet, another preschool intervention, promoting alternative thinking strategies reported improvement of pro-social behaviour, compliance, problem solving and positive feelings [9]. Specialized training for preschool teachers would also provide them with the knowledge and skills to identify children with autism and refer for further investigation of the child [54].

To facilitate early detection and early intervention to support preschoolers’ overall needs, the PLUSS project, based on Bronfenbrenner’s bioecological model [12], was launched in 2019 in Jönköping County, Sweden. The model takes a holistic approach and views development as a complex system of relationships that are affected by the child’s surrounding environment at multiple levels. The closest environment to the child, including interactions with family, preschool, and friends, is the microsystem. The next level is the mesosystem, in which collaboration between different microsystems occurs. Beyond the mesosystem is the exosystem, including local healthcare organisations and local politics. The outermost level of Bronfenbrenner’s bioecological model is the macrosystem, consisting of laws, culture, and economic systems at a national level. Interactions between these different systems affect both the development of the child and their behaviour [12, 13, 14].

The participating preschools are located in municipalities with different socioeconomic status, culture, and population density. The inclusion criteria for children in the PLUSS project are to be at preschool, aged 1.5–4 years, have been referred to a CHC psychologist, and assessed with ESSENCE symptoms (Gustafsson & Korhonen, [29, 30]). Previous research has described preschool teachers’ assessment of behavioural problems, impact on daily life, and engagement among preschool children with ESSENCE symptoms (Gustafsson & Sund Levander, [31, 32]). The intention of the present study is to evaluate a preschool teacher training intervention.

As a framework for the training, we used the applied learning approach to promote active, engaged, and collaborative learning. Applied learning provides opportunities to connect theory and practice, to learn and interact with others different from oneself, and to practise using one’s knowledge and skills. A fundamental aspect of applied learning is to reflect upon the sources and gaps in one’s knowledge and practice, with the intent to improve both the quality of thought and action and the relationship between them. A cornerstone of applied learning is that learning is situated, i.e. it is a process during which the specifics of the context largely determine how people learn. Taking real-world scenarios as a starting point creates a meaningful context, where actors from different environments can reflect and learn together [5].

The theoretical basis for applied learning is constructivist; it suggests that adolescent learning is profoundly social and arises out of the contextual consideration of real-life problems by co-participants. Constructivist theories of learning propose that, fundamentally, individuals learn by constructing meaning through interacting with others and interpreting their environments. The success of applied learning is defined as achieving tangible results for the participating audience or learners, rather than a “grade” that represents an assessment of merits by an individual teacher [15]. We believed that the applied learning approach would enhance collaboration and lead to a mutual sharing of knowledge and experience between parents and preschool teachers, which they could then apply together to the individual child. The PLUSS project used a training intervention consisting of preparatory films and joint workshops for guardians, preschool teachers, and special educators for reflection and learning using real cases of children with ESSENCE symptoms (Gustafsson & Korhonen, [29]). The PLUSS intervention has now been ongoing since 2019 and shows promising results for the child’s everyday functions, engagement and behaviour [32]. However, to further improve the intervention we consider it important to evaluate the preschool teachers’ preferences and experiences of the PLUSS applied learning intervention.

## Aim

The purpose of this research was to study preschool teachers’ preferences and experiences relating to an applied learning intervention to support children with ESSENCE symptoms.

## Methods

### Design

The study has a descriptive design and takes a qualitative approach. It adheres to the Strengthening the Reporting of Observational Studies in Epidemiology (STROBE) guidelines [56].

### Sample

We applied convenience sampling and the inclusion criteria were: responsible preschool teacher/s and special educators with university education of children who had completed the preschool teachers’ training programme. In total, 275 preschool teachers participated in the training programme, and all were invited to take part in the study. Altogether, 164 (60%) accepted the invitation and 20 of these were individually interviewed.

A total of 164 preschool teachers, 98% female, aged 47 *±* 10 years, responded to the questionnaires about their experiences. In addition, of the 164 included, 20 (18 female) were interviewed about their preferences and experiences relating to the workshop. There was no difference in background factors between the total group and the interviewed individuals; age 47 *±* 10 years, working in preschools for 21 *±* 12 years and 85% of them had known the children in their care for more than six months. 92% of all those included had a preschool teacher’s degree, compared to 100% of the 20 who were also interviewed. Nationally, about 40% of preschool teachers have an academic degree (Swedish national Agency for Education, [52]).

### Data collection

Data collection was ongoing during the period May 2019 to December 2024. Directly after completing their training, the preschool teachers filled out an anonymised questionnaire about their experience of the training. They answered the questions either individually or together if they had taken the training jointly for a particular child. Individual interviews were conducted between May 2021 and June 2022, one to two weeks before and three months after the training programme.

### Questionnaire

The questionnaire was developed within the PLUSS project and used to evaluate the preschool teachers’ learning intervention (Gustafsson & Korhonen, [29]). It consists of four items, addressing the experience of the learning intervention as a whole, the lengths of the workshop, whether the learning intervention provided useful ideas for practice, and whether they had gained an increased understanding of the difficulties of the particular child who constituted the scenario in the workshop. The questions about useful ideas and increased understanding offered five response options, ranging from “not at all” to “to a great extent”. For example: “Yes, to a very high degree”; “Yes, to a fairly high degree”; “Yes, to some extent”; “Hard to say”; “No, not at all”. There was also the option of writing a free-text answer to every question.

## Intervention

### Web-based films

The first part of the preschool staff training consisted of seven film modules that were made available online. The modules provided information about the PLUSS project, a child’s normal development and common ESSENCE symptoms, and meeting the child in everyday life. The films included information about aspects such as self-regulation, basic cognition, communication, structure in everyday life, and the role of clarifying pedagogy. The professional lecturers included psychologists from Child Health Care (CHC), speech therapists from the Rehabilitation Centre, and occupational therapists and special educators from Child and Adolescent Rehabilitation (CYH), as well as a researcher, all part of the PLUSS project. Before the COVID-19 pandemic, the first part of the training consisted of a day (four conducted) of lectures that covered the same areas as the film modules. Due to the pandemic, the lectures first switched to being held digitally (two conducted) before the current concept of web-based film modules (17 conducted) described above was implemented in 2021.

### Workshops

For the second part, the workshop, preschool teachers, professionals within PLUSS (those who provided the lectures for the film modules), and representatives from social services met the guardians of the specific child. With this child in mind, the workshop aimed to stimulate joint reflection and discussions of strategies and approaches, e.g. support children with hyperactivity, emotional regulation, peer engagement and play, enhance language. The intention was to stimulate the child’s development both at home and in the preschool environment. An action plan, including follow-up, summarised the child’s difficulties and strengths in everyday structure and follow-up. The workshops were held in person in the county hospital’s lecture halls, except for two digital occasions during the COVID-19 pandemic.

## Data analysis

### Quantitative data

Background data and the four questions in the questionnaire were analysed using descriptive statistics, e.g. percentages and mean *±* SD.

### Qualitative data

The transcribed interviews and the free-text answers from the questionnaire were analysed separately to explore preferences and experiences related to the training. The analysis procedure followed Elo and Kyngäs [21] description of inductive qualitative content analysis. Both authors read all the texts to become familiar with the content. The second author (MSL) then noted meaning units for analysis by open coding, transferred the units to a coding sheet, and grouped them together into preliminary categories. Both authors discussed these categories and combined them into main categories. Finally, after combining the results from the analysis of the interviews and the free answers, a core category emerged as a framework for both preferences and experiences. Tables 1 and 2 illustrate the inductive content analysis process.

**Table 1:** Example of analysis process: Interviews with preschool teachers (*n* = 20) about preferences before the training intervention

Meaning unit	Code	Sub-category	Category/expectations
“I have expectations that it will be rewarding to sit in the same meeting as the guardians and those who are providing the training.” “I’m really curious to hear what they think.” “Share ideas.”	Both the preschool and the parents are involved Hear what the parents think. Share ideas	Share thoughts and experiences with guardians	Deepen and apply professional knowledge about children with special needs
“Gain insight from others’ experiences and gain new knowledge.” “I think it will be an introduction to joint work, which is a very positive thing.”	Share information, ideas, and experiences Learn from the experiences of others	Share thoughts and gain new insights with other professionals	
“That they should feel secure in, well, what we do at the preschool.” “How we should meet the child, both at home and at preschool.”	Input from the network around the child Conveying what the preschool does gives parents confidence How we meet the child	Meeting the child at home and at preschool	Get a picture of and understand the child in different environments

**Table 2:** Example of analysis process: Interviews (*n* = 20) and questionnaire (open-ended answers, *n* = 164) of experiences of the training program with preschool teachers

Meaning unit	Code	Sub-category	Category/Experiences
“And that you have these little meetings with the parents.” “I thought it was valuable when I listened… when I talked to parents…” “I feel that, especially when we got to participate with the parents, that they got to be involved in this, so that they, too, could get tips and ideas on how they can work with the child. And how you can do it, and how we do it in preschool.” **“So valuable to talk to each other about the child. …have time for a deeper conversation about the child.”** **“It’s good to be able to get different perspectives and different focuses on the child.”** **“Together we were able to come up with tools that we can try out practically in everyday life**,** both at home and in** preschool. We highlighted that needs can be different at home and in preschool.”	Sharing tips and ideas Doing the same things together with the parents **Hear about what it’s like at home and at preschool.** **Get a picture of the whole**	Understand the individual child’s difficulties and abilities	Guardians and preschool teachers shared experiences and reflected together about the child
“Thinking and reflecting together with other professions is a value in itself”, it was “very interesting to meet these different people … We were at a meeting with a psychologist and an occupational therapist and a speech therapist, and who they were.” “Gets a little more insight into how to work with children who need special support.” **“New input and new thoughts. Confirmation that the efforts we are already making are good.”** **“It was very positive to meet with guardians and people who work with children in need of support. To discuss together how they work at home**,** but also what we do at preschool.”**	How to work with children who need special support Gain more insights **Share ideas and thoughts.** **In-depth professional knowledge.** **The preschool adapts based on the child’s needs**	Share experiences and learn from other professionals	Collaborative learning and application of professional knowledge about children with special needs

Meaning units and codes from interviews presented in plain text and from questionnaires in bold text

### Ethics

All the research included in the PLUSS project has been approved by the National Ethics Review Board (Registry number 2019–04839 and 2021–05266). Informed consent was obtained in both written and verbal form from all preschool teachers and guardians of the included preschool children, in accordance with the Declaration of Helsinki (WMA, [59]).

Questionnaires and assessment templates were administered on paper and transferred to a computer file. The material was only accessible to PLUSS researchers and patient care staff. The coding template for translation between subjects and their coding was stored in a logbook, which was not accessible to unauthorised persons. The results are presented at group level, and thus no personal information will be recognisable.

## Results

### Preferences before the intervention

Overall, the results illustrate that the preschool teachers wanted and expected the workshop to enable work towards a child-centred approach by sharing experiences and collaboration with the child’s guardians and other professionals, and by deepening their professional knowledge. In the following, the quotes relate to I=interview and Q=questionnaire.

*I always enjoy sharing other people’s experiences... I mean, I always just feel really happy about it. And to gain moreknowledge. To be able to help the children succeed. *(I)

The results revealed that it was essential for the training to contribute to gaining a picture and understanding of the child in different environments. The respondents wanted the training to contribute to a holistic picture through sharing their own and the guardians’ experiences of the child in different environments, and thereby promote collaboration. The special educators also looked forward to more informal contact with the parents.

*I have the expectation that it will be rewarding to sit in the same meeting as the guardians and those who are providing the training.* (I)

*We get to work together well and share ideas and… how we should treat the child*,* both at home and at preschool.* (I)

*And I can feel that I am*,* I’m really curious about that*,* as well as to hear what they think. Of course*,* I talk to guardians when I have to*,* for example*,* write an investigation about a child and so on*,* so I have quite a lot of contact. But then it becomes quite formal. I think this might be a little less formal*,* this particular contact.* (I)

As educators, they also wished to deepen their general professional knowledge about children with special needs and apply it in practice. The preschool teachers emphasised that, although they had extensive experience of children with special needs, they were seeking reassurance that their way of working was the right one. They also wished to gain a deeper understanding of the specific child’s problems, and expressed a desire for new ideas to give the work a fresh start.

*and that I also think correctly in parts*,* as I’ve worked so far in my almost 30 years* (I).

*This is exactly it*,* more knowledge and greater insight into why and how it can happen. And how to work in preschool*,* and bring with me much more competence and information.* (I)

*I want to feel that there would be something*,* something… that gave a little more energy. That would change and that I would have aha*,* yes*,* so*,* yes*,* you can also think that way. Make the impossible possible.* (I)

The preschool teachers stated that their professional focus was on supporting the children and making things easier for them, now and in the future. They wanted concrete suggestions for the team’s methods and tools, like a toolbox, to meet and support the specific child in different situations in the preschool environment.

*So*,* the more tools you have to choose from*,* the easier it is to… find a way that suits this particular child best.* (I)

*Give us knowledge and tools and methods and ideas that we can use and it’s very good that some of the team is involved.* (I)

*I have expectations of getting concrete tools and tips for working with children who need support. And also getting an understanding of how it*,* well*,* how it might work*,* or… Well*,* how children think and work*,* and so on. So*,* I would just like to learn a lot.* (I)

### Experiences after the intervention

The respondents described the training as valuable and said that they had gained general knowledge and insights that benefited their professional competence. They especially appreciated the group sessions, when guardians and professionals shared experiences and reflected together with the focus on a specific child. However, they pointed out that the time they spent in the groups was too short to both reflect and use each other’s knowledge and experiences for the designated child.

*I feel that*,* especially when we got to participate with the parents*,* they got to be involved in this*,* so that they*,* too*,* could get tips and ideas on how they can work with the child. And how you can do it*,* and how we do it in preschool*,* what it can look like for us*,* because I think it’s so important for them*,* also that they gain an insight into how we can have it*,* but also that we also gain an insight into what it’s like at home.* (I)

*So valuable to talk to each other about the child. Very good with reflections between each workshop*,* it meant that we landed on all the thoughts and how we should use the knowledge and how we should work further.* (Q)

*It feels like time goes by very quickly at the different stations*,* which means you don’t really feel finished*,* you’re sometimes left with thoughts that you would have liked to talk more about.* (Q)

The respondents particularly appreciated that the people who participated in these meetings were involved and focused on a specific child with special needs. The conversations provided the opportunity to build a common overall picture of that specific child, to understand their difficulties in everyday life and discuss applicable methods to make things easier for that child. Meeting in person during the group sessions gave a feeling of belonging and working together.

*Taking a look from different perspectives*,* both from preschool*,* child health*,* and the home*,* gives a clearer picture of the child’s entire day*,* which also provides an increased understanding of how the whole affects the child (e.g. emotions*,* sensitivity to demands).* (Q)

*This feels like it holds together more. It becomes a holistic picture. And it’s a better prerequisite for that*,* working with this child.* (I)

*and it feels like it kind of sets the tone a little bit too*,* that we’re working on this together. It’s not just the preschool as its own unit*,* and then there are external contacts*,* and then there are the guardians. But it really becomes this clear*,* now we’re doing this together. I think that’s gold.* (I)

Respondents found their meeting with special preschool teachers valuable for increasing their professional competence by reflecting upon their own experiences and gaining insights through knowledge and shared learning.

*It’s very rewarding to have the opportunity to talk with different professional categories and with guardians. It gives new ideas and makes you think.* (Q)

*Yes*,* after the training*,* I can probably think back to children that I had several years ago*,* like oh*,* why didn’t we do it this way? …why hasn’t anyone said that*,* that I should do it this way*,* before?* (I).

The sessions provided ideas and practical tips to make everyday life easier for the child, both at home and in preschool.

*Together*,* we were able to devise tools that we can try out practically in everyday life*,* both at home and in preschool. We made it clear that needs can be different at home and in preschool.* (Q)

*That you got a lot of new ideas.* (Q)

They also noted that the training confirmed the relevance of the methods and routines used at preschool, but they also gained reflection and new knowledge and ideas about handling specific children.

*I think it’s nice*,* and I think it’s very affirming*,* that the way we think is good to work is good.* (I)

*We feel that we received some new tips to work on but also received confirmation that we’re already on a good track.* (Q)

*I think I’ve gained more knowledge*,* more information and knowledge about what it can be like to be and what it’s about. And*,* then you put it in your bank*,* like. To face situations. … I hope that I kind of … have a greater understanding of how certain things can kind of affect the children and what things we can do can kind of help them*,* and in their everyday lives.* (I)

Some also commented that the course might not have provided much new knowledge, but it is always good to have repetition. Such comments related to the course structure and content, such as requests for more focus on communication and emotion regulation, and more concrete tips to use in different situations. Respondents also expressed a desire for recurring training to strengthen their professional role.

*For me*,* that education is pretty obvious. Since I have… since I’m a trained special education teacher and have worked with it for quite a long time.* (I)

… *if you start from the child we have*,* the focus would have been more on communication and interaction. Then it’s really useful to learn about … conflicts and emotions and such*,* to other children*,* too.* (I)

*Good in the sense that it’s good to hear it several times*,* that you get a “yes*,* that’s it” feeling about things you’ve already heard*,* and that you get new ideas to test.* (Q)

The results also revealed that the films were considered to be good preparation for the applied training workshop and that the preschool teacher and guardians could watch the films and reflect together afterwards. However, the respondents wanted more preparation time at the preschool.

*I think these films were very good*,* the hard part was that they came so late. When you work in a children’s group almost all your time*,* it’s a bit difficult to watch them.* (I)

*I watched them myself and had a meeting afterwards where we reflected together with the guardians. We often had the same experience and thoughts. Good update for me as an educator.* (Q)

The respondents wanted recurring training and more time for collaborative learning and application to practise in the workshop group sessions.

*Competence development at regular intervals. So*,* overall*,* this type of working method*,* or this*,* the input for*,* it probably needs to be refreshed in many places*,* so that it’s*,* this kind of work can be started as early as possible. … Because I think everyone benefits greatly from it*. (I)

*I might have wished to get some more concrete tips from other preschools. Oh well*,* you do that*,* then maybe we can take it with us to my preschool and work with it*,* too.* (I).

### A joint approach to child-centred care

**Fig. 1:**
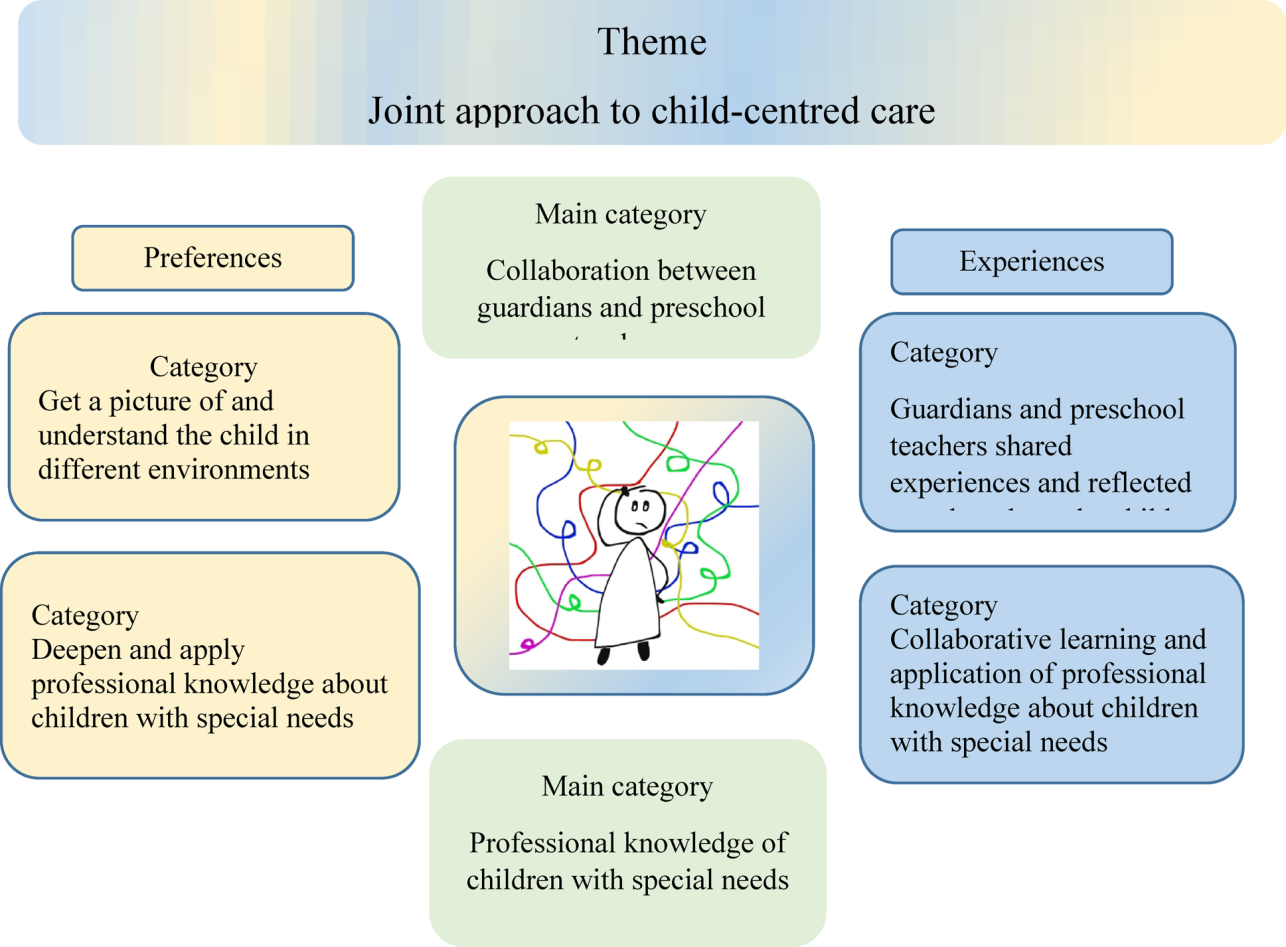
Theme, main category and categories of preferences and expectations by the preschool teachers after training to manage children with ESSENCE symptoms

The qualitative latent analysis of interviews and open-ended questions in the questionnaires revealed that the preschool teachers’ expectations corresponded well to their experiences after completing the training. The main categories *Collaboration between guardians and preschool teachers* and *Professional knowledge about children with special needs* describe their needs when supporting children with ESSENCE symptoms. Together they form the theme *Joint approach to child-centred care*. Figure 1 provides an overview of the preferences and expectations described by the preschool teachers.

### Summary evaluation of the intervention

Almost everyone, 161/164 (98%), thought that the workshop, in its overall structure and content, was very rewarding (*n* = 112, 68%), or quite rewarding (*n* = 49, 30%), while the remaining three respondents (2%) found it to be not very rewarding. Most participants (*n* = 129, 79%) found the workshop to be long enough, while 34 (21%) found the length too short. One person (0.6%) declared the length too long.

**Fig. 2:**
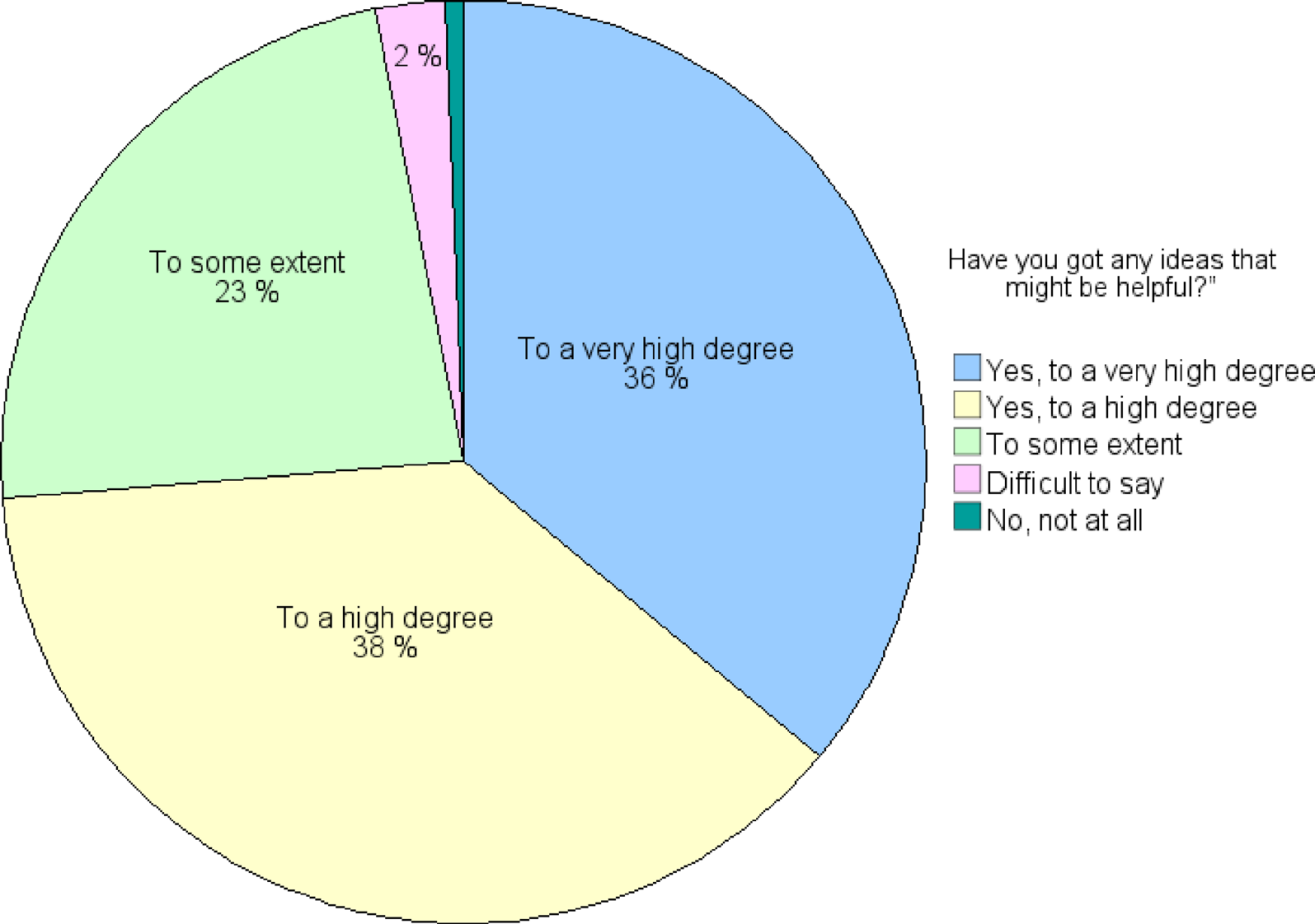
Preschool teachers’ (*n* = 164) responses to: “Have you got any ideas that might be helpful?”

A majority of the preschool teachers experienced that, to a very high degree (*n* = 59, 36%), or to a high degree (*n* = 62, 38%), they got ideas that might be helpful in managing children with ESSENCE difficulties, while 38 individuals (23%) declared this to some extent and four (2%) found it difficult to say. One person (0.6%) declared no, not at all (see Fig. 2).

**Fig. 3:**
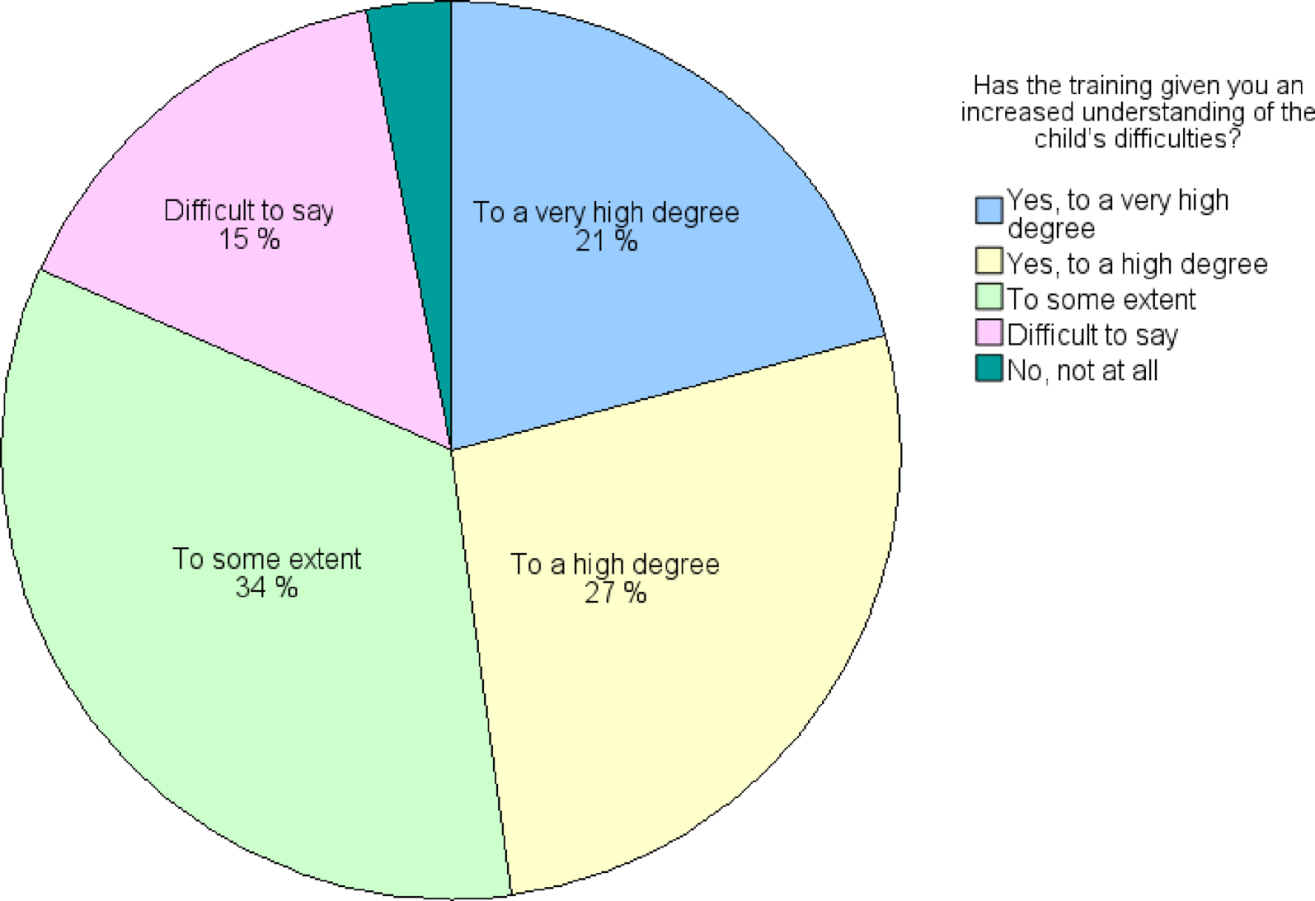
Preschool teachers’ (*n* = 163) responses to: “Has the training given you increased understanding of the child’s difficulties?”

Almost half of the preschool teachers (78/163, 48%) found that the training gave them increased understanding of the child’s difficulties to a very high or high degree, 55 individuals (34%) reported to some extent, while 25 (15%) found it difficult to say, and 3% declared not at all (see Fig. 3).

## Discussion

Overall, the results presented above show that the content of the intervention met the preschool teachers’ expectations. Specifically, the respondents emphasised the value of talking and reflecting together with other professional and guardians about a specific child. Sharing experiences from different contexts provided an understanding of the challenges that the child, and the adults around them, faced in daily life. It also provided insights into the child’s opportunities, abilities, and resources. The meeting with guardians and professionals made it possible for joint learning and the application of professional knowledge about children with special needs.

The results reveal that the preschool teachers wished to support their specific child by collaborating and sharing experiences with the guardians. Such a collaboration contributes to a holistic view of the child and ensures that several of the child’s needs are met through an increased understanding of his/her difficulties, which is in accordance with Swedish school law (SFS, [48]). This is in line with a growing body of research and clinical experience revealing the importance of preschool teachers supporting and collaborating with the families of children with ESSENCE symptoms [17, 24, 27, 50]. Collaboration in the preschool context is supported by the main purpose of health-promotion and intervention, i.e. working with young children to provide learning opportunities and improve their social, cognitive, and adaptive skills during the period of maximal brain plasticity ([23]; AAP, [1]). Early prevention and interventions for preschool children may lead to favourable long-term effects for the children, and lower healthcare costs [34].

Current research has expanded our understanding of neurological developmental processes and the impact of parenting on children’s school readiness [8], For children with ESSENCE symptoms there are different interventions described intended to promote children’s social interaction and emotional and behavioural development [18, 51]. Justicia-Arráez et al., [36], reported positive effects of preschool teachers promoting social-emotional learning in preschools on emotional and communication skills, prosocial behaviors, problem-solving, and social interaction. Yet, another preschool intervention, promoting alternative thinking strategies reported improvement of pro-social behaviour, compliance, problem solving and positive feelings [9]. Specialized training for preschool teachers would also provide them with the knowledge and skills to identify children with autism and refer for further investigation of the child [54].

It is positive that almost all the attending preschool teachers found the training to be rewarding and gave ideas to managing children with ESSENCE difficulties. However, approximately half of the preschool teachers believe that the training had not given them a greater understanding of the child’s difficulties. From the participants’ statements, it is clear that these shortcomings are mainly linked to more preparation time at the preschool before the training, a wish for more time for collaborative learning and application to practise in the group sessions. In addition, individual pre-conceptions, preferences and believs about educational methods might also have impact on how the view the intervention, e.g. Hugh et al., [35] highlights that a prior positive attitude towards an intervention makes preschools prefer this pedagogical method. Nevertheless, the views of the preschool teachers are important for the continued development and improvement of the PLUSS applied learning intervention.

It is clear that the preschool teachers appreciated sharing experiences and reflecting together with guardians about a specific child. This is in line with Avni et al., [6] emphasizing the multiple perspectives for better collaboration between parent and preschool. Collaboration with guardians would also facilitate support of the individual child in every-day life in preschool. Almqvist et al., [4], describe how preschool teachers supported children with special needs by, for example, staying close to the child, giving individual support, and adjusting the preschool environment to facilitate the child. However, according to the macrosystem, i.e. national guidelines (Lpfö, [41]), preschool teachers’ assignment is to make the children’s group function in the first place. Almqvist et al., [4] also state that preschool teachers tend to pay more attention to negative behaviours and are more likely to respond to them. This raises the question of consequences for the individual child when preschool teachers handle negative behaviour by putting the best interests of the group first.

**Fig. 4:**
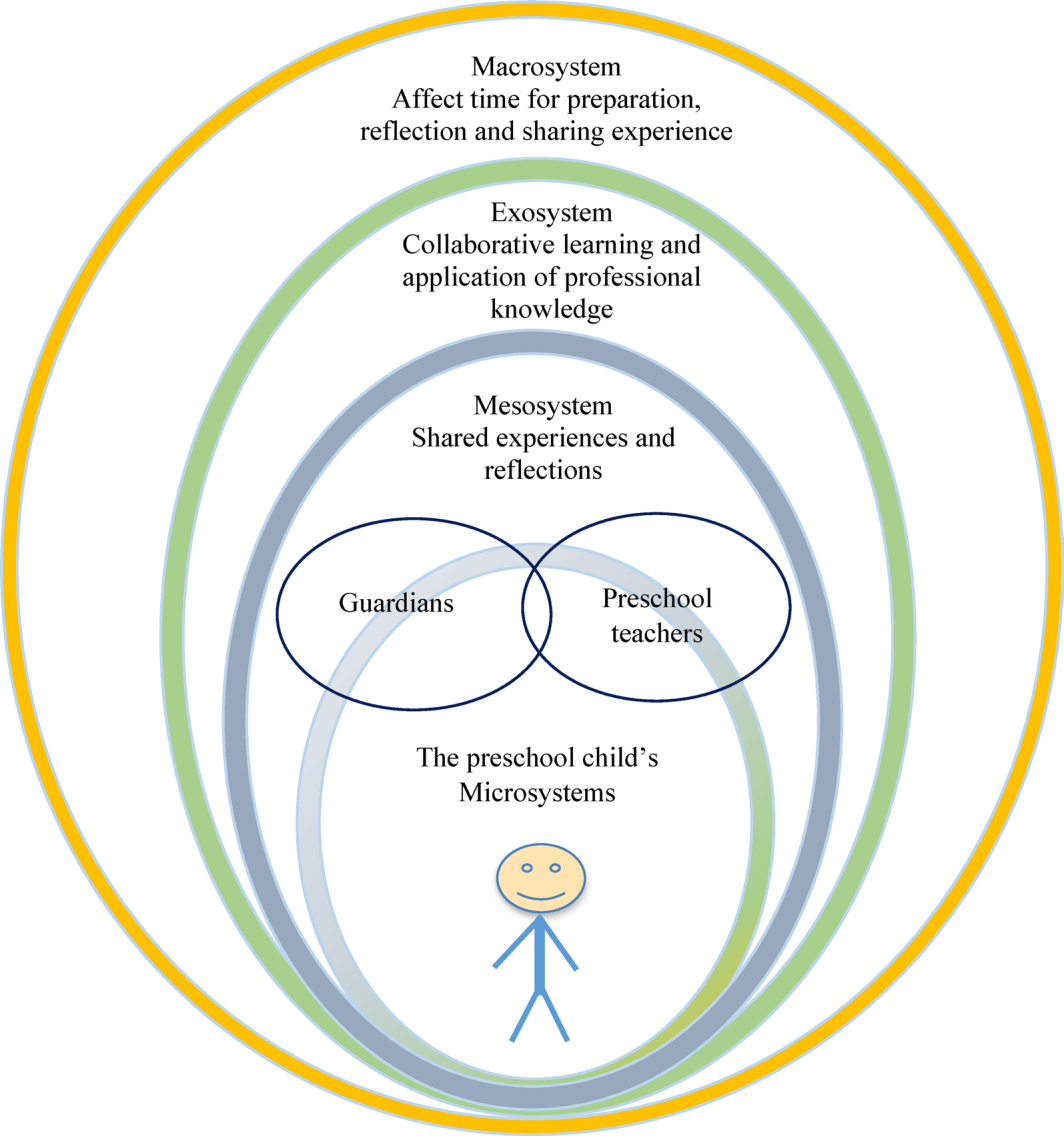
The bioecological model, with the preschool child influenced by the microsystem, mesosystem, exosystem, and macrosystem

As described by Bronfenbrenner [12], the small child is nested within different systems that interact with one another. How well they work together influences the child’s behaviour as well as their developmental and health conditions [27]. According to Bronfenbrenner and Morris [14], the microsystem, which consists of the child’s immediate environment, is the most influential system. In the PLUSS project, this is the guardians and preschool teachers. The mesosystem refers to interactions between the guardians and preschool teachers in terms of shared experiences and joint reflections about the child. In the exosystem, healthcare professionals convey professional knowledge through web-based films and support collaborative learning and application via workshops. Policy and political decisions about priorities in the macrosystem also influence and affect the other three systems. The consequences of this might explain the respondents’ statements about the need for extended time to watch the web-based films, but also increased time for the workshop (see Fig. 4).

In 1980, Knowles et al., [39] coined the term “andragogy” as a pedagogical concept related to adult learning, in contrast to pedagogy, which they described as more adapted to the learning of children and young people. Much of the learning is the same in pedagogy and andragogy, but Knowles believed that adults want to learn more about things that they can use to deal with life based on experienced problems and challenges. Adults often see themselves as responsible for their own lives and the decisions they make and have had time to accumulate different experiences. This leads to differences in their need for knowledge and learning methods. Learning is driven more by internal rewards such as quality of life, self-confidence, and satisfaction in working life. The PLUSS project has adopted this approach in the design of the training intervention. We used applied learning to promote interactions, knowledge, and skills together, and real-world scenarios as a starting point to create a meaningful context, where different professions or different activities can reflect and learn together [5]. We assumed that the workshop would enable the establishment of a friendly atmosphere, promoting trustworthy relationships between guardians and preschool teachers. According to Quaye et al., [45], this enables a holistic view of the specific child as an individual with emotional, physical, psychological, and social needs that extend beyond the needs related directly to the ESSENCE symptoms. The results presented here verify that the training design was successful.

Bierman et al., [8] emphazised preschool-based efforts to help families support the child’s learning at home, with focus on parenting strategies and home learning activities that promote social-emotional and self-regulatory skill development. The found empirical evidence for improvements regarding social skills, impulse control, emotion regulation, and approaches to learning. The interventions, though, reported by Bierman et al., [8] are more generally focused and not specifically for parents of children with ESSENCE symptoms.The preschool teachers wanted to collaborate with guardians to share experiences and reflect together about the child. This is in line with previous research reporting guardians’ desire for tailored interdisciplinary innovations and collaboration within specialist health services for children with multiple referrals to somatic and specialist mental healthcare services [42]. Quaye et al., [45] reported guardians’ wishes for collaboration, trustworthy relationships, and effective communication during their child’s admissions to hospital. The preschool teachers in the present study expressed the same wishes. It is evident that the preschool teachers appreciated the training, which gave them a deeper understanding and insight into each particular child’s needs and strengths. In particular, in line with Ash and Clayton [5], the results reveal that the discussions promoted joint learning and critical reflection when the preschool teachers shared experiences of a specific child with ESSENCE symptoms together with the child’s guardians. Hence, our assumption that applied learning would enhance collaboration and lead to a mutual sharing of knowledge and experience between parents and preschool teachers, which they could apply together to the individual child, was strengthen. From a broader perspective, both the preschool teachers and the guardians expressed the desire for a joint approach to child-centred care.

In accordance with the United Nations Convention on the Rights of the Child (UNCRC), the primary consideration in all matters for preschool teachers is the child’s best interests (UNCRC, [55]). Determining these best interests would ideally involve the combined views of the child, their guardians, and professionals. The child’s best interests, however, are more often elucidated from *a child perspective* than from *the child’s perspective*. The former is an adult understanding of children’s experiences, while the latter represents children’s own understanding and experiences, highlighted in their self-reported narratives [46]. When dealing with all preschool children, we argue that a child’s developmental level and/or ESSENCE symptoms do not hinder the child from expressing his/her own thoughts. Nevertheless, it is crucial that the adults who are present in the child’s microsystem, and see the child in different contexts, work together to understand the individual child’s difficulties and needs in order to provide support in everyday life. That is, they must strive for child-centred care.

A recently published concept analysis established Child and Family Centred Care (CFCC) as an approach in healthcare. The core of CFCC is to place the child at the centre of holistic care, to work in partnership with the child and her/his guardians, and consider the contextual wishes of the child, the family, and the community. Guardians are considered to know their children best and are seen as crucial, both in providing information about their child and in guiding care decisions that align with family values. However, the key to CFCC is that the prevailing needs of the child override the needs of the guardians [60]. Mörelius et al., [44] emphasise that the ability to access, interpret, and apply the best available evidence is key to the practical application of CFCC, and this is also the basis for the design of the PLUSS intervention. In line with the CFCC [60], the intention of the PLUSS project is always to put the needs of the child first, and PLUSS attributes, according to the participants in the intervention, are the same as in the CFCC: collaboration, participation, and communication. Within the CFCC concept, children are active partners alongside their families in all areas that impact upon their health [60].

In the present study, however, the children did not actively participate, but were represented by significant adults in their daily lives. The preconditions for CFCC [60] are also consistent with the findings of the present study; for example, time for preparation, professional knowledge, guidelines, implementer’s perceptions and beliefs, and involvement of family members.

### Limitations and further research

One limitation is that the children’s perspectives were not expressed directly but were conveyed via guardians and preschool teachers. In an upcoming study, we plan to interview children with ESSENCE symptoms to ensure that their voices are heard. There was no initial measurement of preschool teachers’ positioning regarding the educational topics; future research plans to measure this to investigate any possible relationship between preschool teachers’ responses and the effects of the intervention/training. Another limitation is that the COVID-19 pandemic caused changes in both preparations and workshops, which may have affected the interactions between participants.

### Practical implications

The PLUSS project’s learning arrangement of preparatory films and subsequent inter-professional workshops together with guardians enhanced a successful form of applied learning in preschool. The preschool teachers experienced the meeting with guardians and professionals as a joint learning and the application of professional knowledge about children with special needs. Joint reflections and exchange of experiences among guardians and preschool teachers, with the focus on a specific child, could be part of every preschool’s regular activities. This requires that organisational and structural conditions, such as time, must be met.

## Conclusion

The findings presented here add insights about collaborative learning, the application of professional knowledge, and guardians and preschool teachers sharing experiences and reflecting together to support each child with ESSENCE symptoms in their everyday life. In this way, the study also contributes to the improvement and application of Child and Family Centred Care in practice.

## Data Availability

All data generated or analysed during this study are included in this published article. Additional data are available from the corresponding author on reasonable request.
